# Growth Superiority and Genetic Characterization of the Hybrid from Female Ussuri Catfish (*Pseudobagrus ussuriensis*) and Male Longsnout Catfish (*Leiocassis longirostris*)

**DOI:** 10.3390/ani14243617

**Published:** 2024-12-15

**Authors:** Minghua Xie, Yulin Zhou, Yi Gong, Min Liu, Peng Zhen, Zhi Li, Li Zhou, Jianfang Gui, Zhongwei Wang

**Affiliations:** 1 College of Fisheries and Life Science, Dalian Ocean University, Dalian 116023, China; xieminghua@ihb.ac.cn; 2 State Key Laboratory of Freshwater Ecology and Biotechnology, Hubei Hongshan Laboratory, The Innovation Academy of Seed Design, Institute of Hydrobiology, Chinese Academy of Sciences, Wuhan 430072, China; zhouyulin@ihb.ac.cn (Y.Z.); gongyi@ihb.ac.cn (Y.G.); liumin@ihb.ac.cn (M.L.); zhenpeng@ihb.ac.cn (P.Z.); lizhi@ihb.ac.cn (Z.L.); zhouli@ihb.ac.cn (L.Z.); 3 University of Chinese Academy of Sciences, Beijing 100049, China

**Keywords:** *Pseudobagrus ussuriensis*, *Leiocassis longirostris*, crossbreeding, phenotype, molecular marker

## Abstract

Crossbreeding is a traditional breeding technique and has been performed successfully in many fish species. However, distant hybridization between different genera is hard to be successful because of reproductive isolation. In this study, diploid hybrids (PL) were successfully derived from the hybridization of Ussuri catfish (*Pseudobagrus ussuriensis*, PU, ♀, 2n = 52) and longsnout catfish (*Leiocassis longirostris*, LL, ♂, 2n = 52). And the morphological data, external frame parameters, chromosomal karyotypes, DNA content measurement, mitochondrial DNA control region, and species-specific marker identification were applied to investigate the traits and genetic characterization of the PL hybrid offspring and their parents. Both quantifiable traits and shape frame parameters of the PL hybrid offspring were revealed to be intermediate between those of their parents; however, cluster analysis showed that their external morphology was more in favor of the maternal PU. The growth comparisons showed that the hybrids had significant growth advantages over maternal PU. Based on karyotype patterns, DNA contents and mitochondrial DNA, the hybrid origin and maternal inheritance of hybrid offspring were further confirmed. According to the sequence variations identified from the genome sequences of the two catfish species, one species-specific marker was developed to distinguish the PL hybrid offspring and their parents. Therefore, this study provides a successful case for intergeneric hybridization and hybrid superiority, and the PL hybrid shows promise for commercial application, pending further studies into its husbandry, health, and welfare with larger populations of fish.

## Simple Summary

Crossbreeding is a traditional breeding technique that usually leads to significant hybrid advantages in the offspring. In this study, we crossed the female Ussuri catfish with the male longsnout catfish and successfully produced diploid hybrid offspring with a significant growth advantage. The external morphology of the hybrid was intermediate between that of the parents; however, the hybrid could not be judged only by morphology. Therefore, this study aimed to develop a DNA molecular marker that can quickly and accurately distinguish hybrid offspring from parents. The results of this study provide successful examples of intergeneric hybridization, and the hybrid offspring have potential commercial value in the future.

## 1. Introduction

Distant hybridization can transfer the genome of one species to another, resulting in progeny changes in genotypes and phenotypes. In particular, at the phenotype level, hybridization often leads offspring to show advantages in growth, survival rate, productivity, feeding ability, and disease resistance [1,2,3,4,5,6,7,8]. Crossbreeding is also characterized by a short cultivation cycle, and generally, the hybrid superiority can be expressed in the next generation, which helps improve economic efficiency in aquaculture [9,10]. For example, hybrid snakehead shows higher survival, and better stress resistance and growth performance compared to its parents; therefore, the most common cultured snakehead species is hybrid snakehead (*Channa maculate* ♀ × *C. argus* ♂) [11]. Interspecific hybrid catfish *(Ictalurus punctatus* ♀ × *I. furcatus* ♂) demonstrated potential heterotic growth, and now represent 70% of the catfish production in the United States, replacing channel catfish (*I. punctatus*) with hybrid catfish [12].

Ussuri catfish (*Pseudobagrus ussuriensis*, PU) is one of the most important indigenous species in China and shows high potential for aquaculture exploitation in China due to its significant advantages, including excellent taste, high market value, high resistance against diseases, and the availability of reproduction technology [13,14,15,16]. Longsnout catfish (*Leiocassis longirostris*, LL) is a semi-migratory and commercially important freshwater species endemic to China, especially in the Yangtze River and Pearl River [17,18]. In recent years, the demand and commercial value of LL has been increasing rapidly due to its nutritional value and flavor with high relative content of unsaturated fatty acids and oleic acid [19,20]. Therefore, PU and LL were used for genetic improvement. Compared with longsnout catfish, a large-bodied Bagridae catfish, Ussuri catfish has a much smaller body size. Therefore, longsnout catfish is an appropriate catfish for the genetic improvement of Ussuri catfish. It has been found that the growth rate and muscle mass of the F_1_ generation of a cross between yellow catfish (*Pelteobagrus fulvidraco*) (♀) and LL (♂) were improved [21]. Similarly, the F_1_ progenies of crosses between PU (♀) and *P. fulvidraco* (♂) had better growth rate and fattening compared to self-crosses [22,23]. However, no artificial hybridization between PU and LL has been conducted.

In this study, we performed hybridization between female PU and male LL, producing the hybrid offspring PL. Morphological features and growth traits between the offspring and their parents were compared. Then, the genetic characterization of the offspring was revealed by chromosomal karyotypes, DNA content, a mitochondrial DNA control region sequence, and a species-specific nuclear marker. Consequently, the hybrid with a growth advantage may serve as a new aquaculture variety in the future.

## 2. Materials and Method

### 2.1. Animals and Ethics Statement

PU and LL were reared to be sexually mature in the National Aquatic Biological Resource Center (NABRC, Wuhan, China). All experiments and animal treatments were carried out according to the principles of Animal Care and Use Committee of the Institute of Hydrobiology, Chinese Academy of Sciences. All the catfish individuals used in this study were anesthetized with 100 mg/L MS-222 before dissection.

### 2.2. In Vitro Reproduction and Progeny Rearing

Five male and ten female individuals of both PU and LL catfish were randomly selected to perform the crossing by artificial spawning using hormone injecting. Mature female PU was firstly injected with a dose of 2 μg/kg LHRH-A2 and 2 mg/kg pituitary gland (PG), followed by a second injection with a dose of 5 mg/kg DOM, 5 μg/kg LHRH-A2 and 2 mg/kg PG 12 h later. Mature male LL and PU were injected with half the dose given to females, using one injection at the same time as the second injection given to female PU. Then, half of the mixed eggs from three female PU individuals were fertilized with sperm from one male LL individual. Another half of the mixed eggs were fertilized with sperm from one male PU individual. After fertilization, fertilized eggs were adhered to nylon meshes, then transferred to incubation pools with sufficient oxygen at 24–26 °C until larvae hatched. For each of the two groups, 1000 embryos were selected to be counted for the fertilization rate and hatching rate. Subsequently, the same number of fries were counted and reared in 3 m^3^ concrete tanks under identical culture conditions.

### 2.3. Measurement of Morphological Indicators

**Figure 1:**
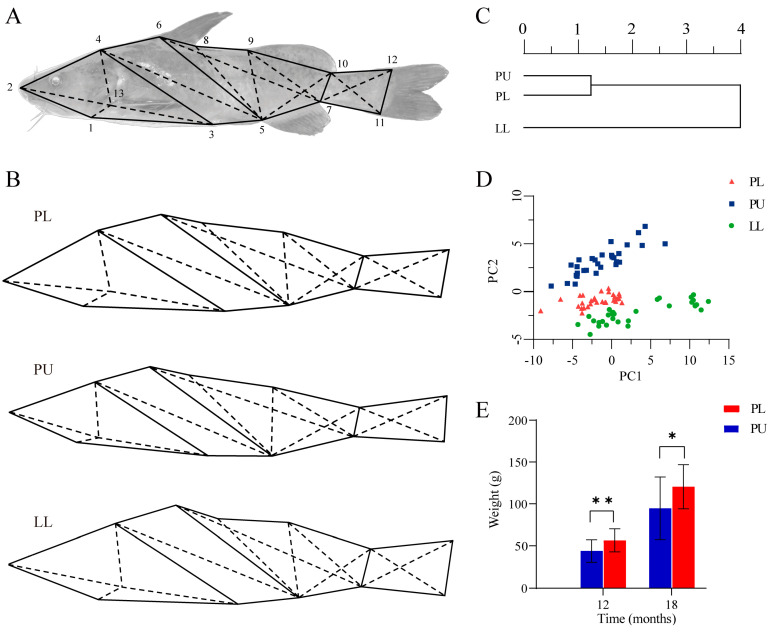
Comparison of appearance, morphology, and growth properties between the PL hybrid offspring and their parents. (**A**) Measured drawing of fish body frame structure. There are 13 coordinate points, where D1−2 indicates the distance between coordinate points 1 and 2, and so on. Among them, 1: the lower end of the mandible; 2: the front end of the snout; 3: the front end of the ventral fin; 4: the upper end of the maxilla; 5: the front end of the anal fin; 6: the front end of the dorsal fin; 7: the rear end of the anal fin; 8: the rear end of the dorsal fin; 9: the front end of the adipose fin; 10: the rear end of the adipose fin; 11: ventral origin of caudal fin; 12: dorsal origin of the caudal fin; 13: the front end of the pectoral fin. (**B**) Body shape truss network of PL, PU, and LL. (**C**) Hierarchical dendrogram of PL, PU, and LL. (**D**) Scatter diagram for PC1 and PC2 plot of PL, PU, and LL. (**E**) Comparison of growth performance between PL, PU, and LL. Significant differences in weight were marked with asterisks; * indicates *p* < 0.05, ** indicates *p* < 0.01.

One hundred randomly selected fish from each group, PL, PU, and LL, were reared in 3 m^3^ tanks and fed twice a day until the test fish were apparently satiated. During the 12 months of culture, thirty fish individuals were randomly selected from PU, LL, and their offspring group to measure their morphological indices, including the whole length (WL), body length (BL), body height (BH), head length (HL), snout length (SL), eye diameter (ED), interorbital width (IW), caudal peduncle length (CPL), and caudal peduncle height (CPH). In addition, 27 frame data were determined based on the schematic diagram of the frame structure (Figure 1A). In order to reduce the influence of different fish body sizes on the test results, the measurable traits were corrected by ratio treatment. Nine morphological ratio data were calculated, including WL/BL, BL/BH, BL/HL, HL/SL, HL/ED, HL/IW, BL/CPL, BH/CPH, and CPL/CPH. Hybrid index (HI) was calculated according to the following formula [24,25]:

$$HI=100\times \left(H_{i}-M_{i1}\right)/(M_{i2}-M_{i1})$$

where H_i_ is the mean value of hybrid traits, M_i1_ is the mean value of maternal traits, and M_i2_ is the mean value of paternal traits. SPSS 24 statistical software was used to perform cluster analysis and the principal components and differences among three groups were analyzed with one-way ANOVA.

### 2.4. Assessment of Growth Performance

When the fries grew to about 2 g, they were randomly selected from the PL and PU groups and placed into 3 m^3^ of concrete tanks for growth experiments, with a culture density of 100 fish/tank in one group; thus, three parallel groups were constructed. All fish were kept under the same conditions and fed twice a day, each time until the test fish were visibly satiated, using a commercial diet containing 43% crude protein (42.00% fish meal, 11.50% wheat flour, 10.00% soybean meal, 9.80% rapeseed meal, 9.60% cottonseed protein, 5.39% vitamin and mineral premixes, 3.50% fish oil, 3.50% soybean oil, 2.50% sodium carboxymethyl cellulose, 2.00% Ca(H_2_PO_4_)_2_, 0.11% choline chloride, and 0.10% ethoxyquinoline). The water temperature in the cement tank was maintained in the range of 24–28 °C, the dissolved oxygen level was above 5 mg /L, and other parameters were kept within the appropriate range (including ammonia nitrogen content < 0.05 mg/L; pH 7.4–8.0). Ten fish from each group were randomly selected and weighed (30 from PL and 30 from PU) at 12 and 18 months of culture. The weight of the fish was measured by an electronic scale (accurate to 0.1 g). The mean and standard deviation of the growth data of each group were calculated using SPSS software and the difference was tested for significance.

### 2.5. Measurement of DNA Content via Flow Cytometry

Blood was aspirated from the clipped fins of PL, PU, and LL individuals. The reaction was carried out using 1 μL of blood mixed with 200 μL of CyStain^®^ DNA 1 step solution at 4 °C using the procedure described in the kit instruction manual (Sysmex Corporation, Kobe, Japan) as described previously [26]. The DNA content measurements were then performed using PB450-A by Cytoflex S Flow Systems (Beckman Coulter, Inc., Brea, CA, USA).

### 2.6. Preparation of Chromosome Spreads

Chromosome preparations were carried out on the kidney tissues of PL, PU, and the LL hybrid offspring according to the previously described method [27]. Phytohemagglutinin (PHA) was injected into each fish; then, chromosomal preparations were obtained from kidney cells, the cells were treated with hypotonic solution at 37 °C for 1 h, and then 2 mL of fixative (methanol/acetic acid = 3:1) was added and fixed for 30 min, during which the fixative was replaced every ten minutes. Finally, the cells were resuspended in the fixative, spread on pre-cooled slides, air-dried, and stained with Giemsa’s stain. In order to count the plurality of the chromosome numbers of the three species, 100 clear mid-chromosome division phase cells were selected in each fish and photographed under a microscope. Well-dispersed, high-quality images of the metaphase phases were selected for karyotyping according to the criteria stated in [28].

### 2.7. Comparison of Mitochondrial DNA Control Region

Ten hybrid PL, three PU, and one LL offspring were anesthetized with 100 mg/L MS-222. Then, small pieces of the caudal fins were cut with the front end of alcohol-sterilized scissors for DNA extraction in a process that reduces damage to the fish. The DNA was extracted with a DNA extraction kit (Promega, Madison, WI, USA). Based on the mitochondrial genomes of PU (GenBank: NC_020344.1) and LL (GenBank: NC_014586.1), PCR reactions were performed to amplify the mitochondrial control region in the DNA of the PL, PU, and LL samples using the primer pairs of DL1 (5′-ACCCCTGGCTCCCAAAGC-3′) and DH2 (5′-ATCTTAGCATCTTCAGTG-3′). The amplification products were sequenced. DNAMAN 8 software was applied to compare the sequences, base contents and variant sites were calculated using MEGA 11 software, and haplotypes and polymorphic site distribution were calculated using DNAsp 6 software.

### 2.8. Development and Validation of Species-Specific DNA Marker

Based on the genomes of PU (GenBank: PRJNA810258) and LL (GenBank: PRJNA692071), the chromosome sequences of PU and LL were aligned using minimap2 software (https://github.com/lh3/minimap2, accessed on 21 June 2024). Collinearity and rearrangement analysis was performed using NGenomeSyn (https://github.com/hewm2008/NGenomeSyn, accessed on 21 June 2024). Based on the above results, one gene with long deletions was selected to design the specific marker using Primer 5 according to the variation regions between PU and LL.

Genomic DNA was extracted from each of the ten fin tissues of PL, PU, and LL using a Wizard^®^ Genomic DNA Purification Kit according to the manufacturer’s instructions. DNA quality and concentration were assessed by agarose electrophoresis and UV spectrophotometry, respectively. Parents and offspring were distinguished by PCR amplification using the designed species-specific primer (F: 5′-GCCAAACCCATCTCCACATAT-3′ and R: 5′-GGCGTAAAGCCTCACATCAAA-3′).

## 3. Results

### 3.1. Fertilization Rates and Hatching Rates

We set PU (♀) × LL (♂) as the hybrid group and PU (♀) × PU (♂) as the control group. Fertilization rates and hatching rates were counted for both the hybrid and control groups. High fertilization rates were observed in both groups, with values of 89.6% in the hybrid group and 90.5% in the control group. Similarly, the high hatching rates of 85.3% in hybrid and 86.6% in control group were also observed, indicating that there were no significant differences between the two groups in fertilization and hatching rates.

### 3.2. Morphological Differences Between the PL Hybrid and Their Parents

As shown in Table 1, a total of eight quantifiable trait ratios showed significant differences between PU and LL (*p* < 0.05), which accounted for 88.89% of all quantifiable trait ratios. In particular, HL/IW and BH/CPH of the PL hybrid were significantly different from PU but not LL. Instead, HL/SL and HL/ED of the PL hybrid were significantly different from LL but not PU. Regarding the hybrid index (HI), BH/CPH (>100), in particular, exhibited super-paternal deviation among all nine quantifiable traits. The values of BL/HL, HL/IW, BL/CPL, and CPL/CPH in the PL hybrid showed a favored paternal dominance, while those of WL/BL, BL/BH, HL/SL, and HL/ED showed a favored maternal dominance. The mean value of the HI was 51.38, which indicated that the ratios of quantifiable traits of the PL hybrid were generally intermediate between the parents.

Furthermore, frame analysis also revealed some differences between the PL hybrid and their parents (Figure 1B). In brief, PU had a shorter head, a distinctly extended posterior carapace, and a rectangular caudal morphology with an overall elongated shape. However, LL had a broader head with a protruding snout end, a broader mid-body, and a broader and trapezoidal bias in the caudal region. As for the PL hybrid, it had a broader head, similar to its parents, but showed a less prominent snout end, a wider midsection, and an elongated caudal peduncle, which suggested that the overall shape of the PL hybrid was intermediate between those of the parents.

A dendrogram of the cluster was constructed based on the Euclidean distance between the parents and the PL hybrid by SPSS (Figure 1C). The PL hybrid clustered with the maternal PU at first, and then both of them clustered with the paternal LL group, indicating that the PL hybrid was more similar to the female PU in external morphology than the male LL. Further, the contribution rates of four principal components from 36 traits of the PL hybrid and its parents are shown in Table 2. The contribution rates of principal component 1 (PC1) was 56.20%, and the factor loading values of D3-13, D6-7, and D5-9 were 0.961, 0.950, and 0.941, respectively. PC1 mainly reflected the position information of the pectoral and ventral fins, dorsal fin, adipose fin, and anal fin, suggesting that the phenotypic differences between parents and hybrid offspring mainly occurred in midsection. The factor loading values of BL/HL and CPL/CPH were over 0.8, which showed that PC2 mainly represented a contribution in the head and tail characteristics of the variations. The PL hybrid and its parents were divided into three groups, each with its own morphological characteristics according to the scatter diagram for PC1 and PC2 (Figure 1D).

### 3.3. Growth Superiority of the PL Hybrid over the Maternal PU

Comparisons of growth traits were performed in two different growth stages (Figure 1E). At 12 months, the mean weight of the PL hybrid was 56.35 ± 13.66 g, which showed a significant growth advantage of 28.45% over maternal PU, with a mean weight of 43.87 ± 13.4 g (*p* < 0.05). At 18 months, the mean weight of the PL hybrid was 120.43 ± 26.31 g, which was significantly greater than that of PU (94.55 ± 37.6 g), with a difference of 27.37% (*p* < 0.05). It also indicated that the growth rate before 12 months was smaller than that during the stage from 12 to 18 months in both the PL hybrid and PU. It also indicated that the growth rate before 12 months was smaller than that during the stage from 12 to 18 months in both the PL hybrid and PU.

### 3.4. DNA Contents and Chromosome Comparisons of the PL Hybrid and Their Parents

**Figure 2:**
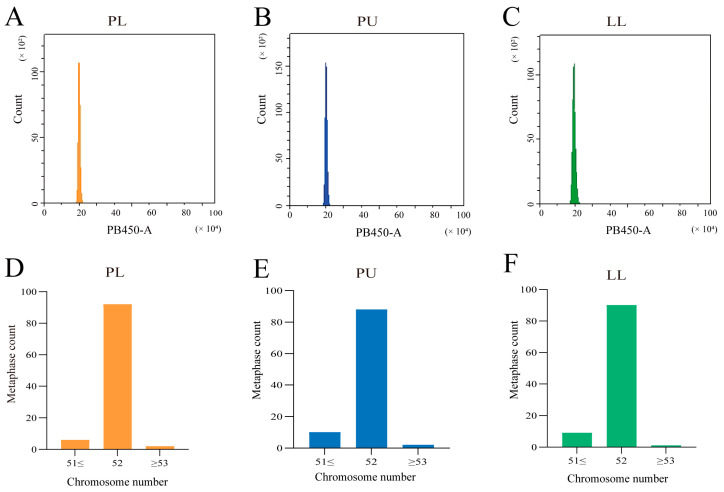
Histogram of DNA content of blood cells and examination of chromosome number in the PL hybrid and their parents. (**A**) The mean DNA content of the PL hybrid. (**B**) The mean DNA content of PU. (**C**) The mean DNA content of LL. (**D**) Distribution of chromosome numbers in 100 metaphases of the PL hybrid. (**E**) Distribution of chromosome numbers in 100 metaphases of PU. (**F**) Distribution of chromosome numbers in 100 metaphases of LL.

**Figure 3:**
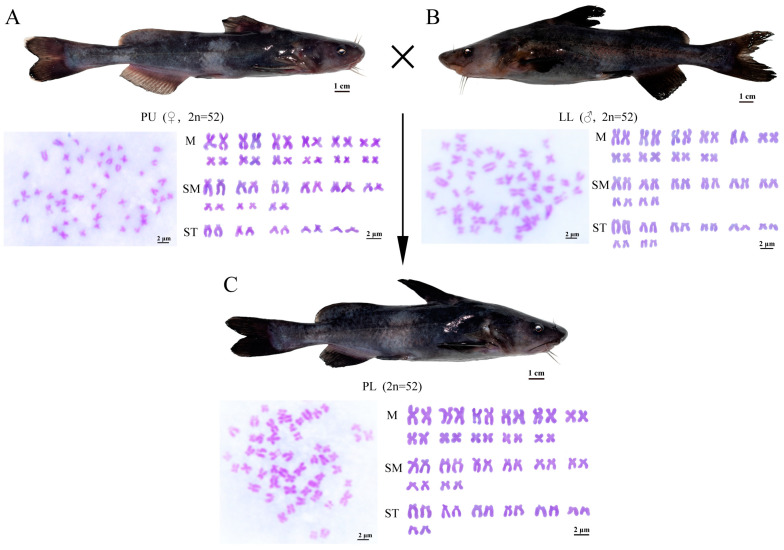
Formation procedure, chromosomal traits, and appearance of the PL hybrid and their parents. (**A**) The appearance, chromosome number, and chromosome karyotype of PU. (**B**) The appearance, chromosome number, and chromosome karyotype of LL. (**C**) The appearance, chromosome number, and chromosome karyotype of the PL hybrid.

Based on the DNA content peaks (Figure 2A–C), the mean PB450-A values for DNA content of the PL hybrid, PU, and LL cells were 20.36 × 10^4^, 20.54 × 10^4^, and 20.17 × 10^4^, respectively, which indicated that the DNA content of the PL hybrid was the median of PU and LL. To determine the chromosome number of the PL hybrid and their parents, the best 100 metaphases from five individuals of the three species were examined. As shown in Figure 2D–F, a total of 88.0%, 90.0%, and 92% of the counted metaphases had 52 chromosomes in PL, PU, and LL, respectively, suggesting that the model chromosome number of both the PL hybrid and its parents was 52. Further, the chromosomal karyotype analysis was conducted, and the karyotype formulas of PU and LL were 2n = 24m + 18sm + 10st and 2n = 20m + 16sm + 16st, respectively (Figure 3A,B). In addition, 92.0% of the chromosomal metaphases supported that the PL was a diploid hybrid with a karyotype of 2n = 22m + 16sm + 14st (Figure 3C).

### 3.5. Identical Mitochondrial DNA of the PL Hybrid with Maternal PU

**Figure 4:**
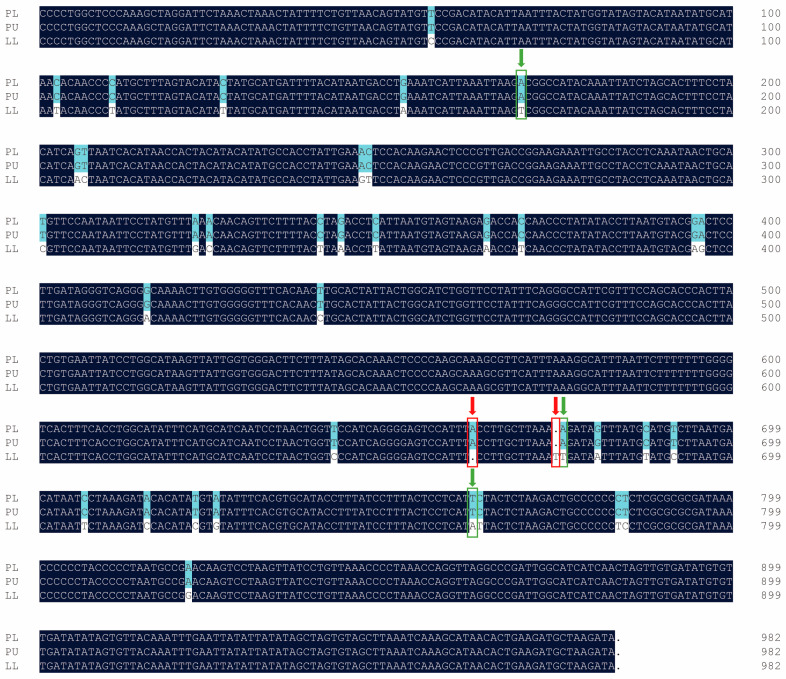
Variant nucleotide sites in the mitochondrial DNA control region of the PL hybrid offspring and their parents. The red arrow indicates the single nucleotide indels; the green arrow indicates the substitution sites.

Mitochondrial D-loop fragments with a 982 bp length were amplified by PCR in both the PL hybrid offspring and their parents. A total of 38 variation sites (3.87%) including 3 substitutions, 2 single nucleotide Indels, and 33 transitions were detected between the two parents (Figure 4). The sequence of the PL hybrid was completely identical to that of the maternal PU, while it differed from that of the paternal LL. The frequency of gene bases in the mitochondrial D-loop was calculated. The guanine-cytosine (GC) content (38.6%) of the complete sequence was lower than the adenine-thymine (AT) content (61.4%) in PU and the PL hybrid, while it was 38.3% and 61.7% in LL, showing a specific AT base preference.

### 3.6. Specific Marker Development and PL Hybrid Identification

**Figure 5:**
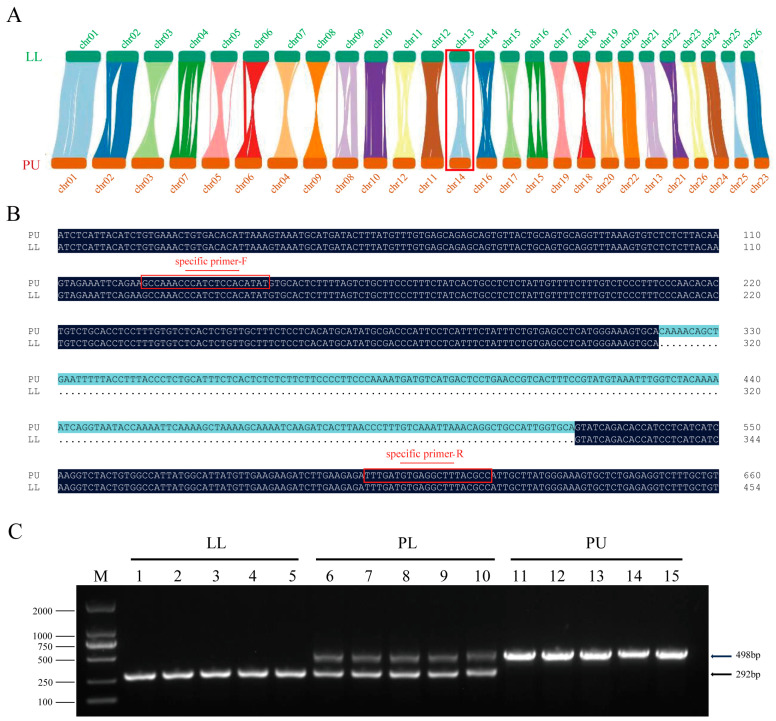
Species-specific DNA marker of the PL hybrid offspring and their parents. (**A**) Chromosome sequence alignment and collinearity analysis between PU and LL. (**B**) Sequence alignment between PU and LL. (**C**) Electrophoresis results of PCR products using specific primers in PL, PU, and LL.

Based on the genomes of PU (GenBank: PRJNA810258) and LL (GenBank: PRJNA692071), the chromosomal collinear synteny between the two catfish species was evaluated (Figure 5A). The chromosomes of these two species were highly syntenic to each other, indicating relatively conserved karyotypes. According to the genome sequence variations between PU and LL, one fragment with 206 bp deletions in the actin binding LIM protein family member 3 (*ablim3*), located in Chr 13 of LL and Chr 14 of PU, was identified; then, a pair of specific primers was designed to develop the species-specific marker (Figure 5B). As expected, only one 498 bp band was observed in PU, and only one 292 bp band was observed in LL by PCR, using the specific primer pair, indicating that this primer pair could be applied to distinguish the two catfish species efficiently. Subsequently, five samples from the hybrid and each of the parents were collected to validate this species-specific primer. Two bands with 498 bp and 292 bp were obtained in PL hybrid (Figure 5C), indicating that the PL hybrid inherited them from both maternal PU and paternal LL.

## 4. Discussion

External morphological characteristics are among the most important characteristics of fish, influenced by both the environment and genetics, and they also are the most intuitive and important basis for fish classification [29,30]. Because data on fish morphological characteristics are easily available and simple to manipulate, the application of statistical methods to reveal phenotypic traits is still an important means for excellent species breeding [31,32,33]. In addition, frame analysis and morphometric analysis have been effectively combined in germplasm identification [25,34,35,36]. In the present study, offspring were obtained through distant hybridization between PU and LL, and significant phenotypic changes were revealed by the morphological trait and shape frames. Furthermore, the morphological traits of offspring were intermediate between PU and LL. Therefore, we support that the offspring resulted from the hybridization of PU and LL, rather than the formation of gynogenesis or androgenesis. It is worth noting that our produced PL hybrid offspring were all observed to be female, and their fertility requires further investigation because similar fertility changes and reproduction mode transitions have been observed in gibel carp (*Carassius gibelio*) [37,38].

The hybrid of the F_1_ generation is frequently characterized by heterosis, such as excellent growth performance, higher survival, and higher resistance to the environment and diseases [39,40]. Genetically based mechanisms responsible for the heterosis of F_1_ hybrid in fish have recently been investigated. For example, the regulatory network between DNA methylation and miRNAs was uncovered and the mechanisms of the heterosis of growth traits were revealed in allotriploid fish [41]. The upregulation of genes involved in the calcium signaling pathway in muscles is the major contributor to the growth superiority in the Grouper hybrid of female *Epinephelus fuscoguttatus* and male *E. lanceolatus* [42]. In catfish species, the F_1_ hybrid of female channel catfish (*I. punctatus*) × male blue catfish (*I. furcatus*) have been the primary driver of US catfish production in recent years because of their superior growth, survival and carcass yield, and fatty acid metabolism/transport pathways, which may explain the faster growth of the F_1_ hybrid [43,44]. In this study, the hybrid of female PU and male LL with a significant growth advantage was created successfully. Both PU and LL are sexually dimorphic, and the males have a larger body size than the females. The all-female PL hybrid offspring grow faster than both female and male PU, which supports the fact that PL has a growth advantage due to hybrid vigor. However, its genetic mechanisms for heterosis need further investigation. The excellent growth trait can shorten the culture cycle, which can help reduce the culture cost and avoid aquaculture risks in aquaculture practice.

Hybrid offspring usually show similar morphological characteristics to their parents, which makes it difficult to accurately distinguish them solely based on morphological characteristics. Therefore, some genetic markers, such as mtDNA, 5S rDNA, and SSR, have been developed to reveal the origin of hybrid offspring and distinguish the offspring from their parents [45,46,47]. Mitochondrial DNA, as a cytoplasmic DNA marker, follows strict matrilineal inheritance in most eukaryotic organisms, and is characterized by a high rate of evolution and minimal recombination [48]. In addition, due to the development of genome sequencing and analysis technology, a method of species-specific DNA marker developed by genome sequences was exploited in *P. fulvidraco* [49]. In this study, a similar mtDNA sequence was detected in hybrid offspring and PU, which suggested its strict maternal inheritance characteristics. Specific markers developed in this study can identify PL and PU stably and efficiently, which not only help overcome the difficulties of morphological methods and facilitate the management of aquaculture production, but they may also play an important role in germplasm resource conservation and genetic breeding.

## 5. Conclusions

In this study, we successfully produced a diploid hybrid from the hybridization of PU and LL. The phenotypic and genetic differentiation of the parents and the PL hybrid were investigated using morphological data, external frame parameters, chromosomal karyotypes, DNA content measurements, the mitochondrial DNA control region, and species-specific marker identification. The results of the study provide a successful case of intergeneric hybridization and a theoretical reference for similar studies in the future. The PL hybrid offers promise for commercialization, pending further studies on their rearing, health, and welfare for future development.

## Figures and Tables

**Table 1:** Comparative analysis of measurable characteristics and the hybridization index of the PL hybrid and their parents.

Items	PL	PU	LL	*p*-Values	Hybrid Index
WL/BL	1.13 ± 0.01 ^b^	1.11 ± 0.02 ^c^	1.16 ± 0.02 ^a^	<0.001	40.00
BL/BH	6.03 ± 0.16	6.10 ± 0.84	5.82 ± 0.32	0.106	25.00
BL/HL	3.86 ± 0.14 ^b^	4.42 ± 0.15 ^a^	3.63 ± 0.13 ^c^	<0.001	70.89
HL/SL	2.81 ± 0.22 ^a^	2.86 ± 0.25 ^a^	2.58 ± 0.11 ^b^	<0.001	17.86
HL/ED	8.02 ± 0.57 ^b^	7.98 ± 0.71 ^b^	11.83 ± 1.34 ^a^	<0.001	1.04
HL/IW	2.48 ± 0.16 ^a^	2.24 ± 0.24 ^b^	2.59 ± 0.16 ^a^	<0.001	68.57
BL/CPL	4.99 ± 0.17 ^b^	4.68 ± 0.21 ^c^	5.24 ± 0.2 ^a^	<0.001	55.36
BH/CPH	2.25 ± 0.11 ^b^	2.71 ± 0.4 ^a^	2.27 ± 0.13 ^b^	<0.001	104.55
CPL/CPH	2.72 ± 0.16 ^b^	3.48 ± 0.42 ^a^	2.52 ± 0.19 ^c^	<0.001	79.17
mean					51.38

Note: Values are presented as the means ± SD. n = 30. Different superscripts at the top right of the values indicate they are significantly different (*p* < 0.05).

**Table 2:** Loadings and cumulative contribution rates of four principal components from the thirty-six traits of the PL hybrid and their parents.

Morphological Ratio Parameter	Principal Components			
	PC1	PC2	PC3	PC4
WL/BL	0.121	−0.715	−0.299	0.300
BL/BH	−0.360	0.033	0.755	0.383
BL/HL	−0.197	0.916	0.095	0.037
HL/SL	−0.171	0.458	0.412	−0.241
HL/ED	0.654	−0.518	−0.221	0.302
HL/IW	0.146	−0.641	0.467	0.298
BL/CPL	0.315	−0.737	−0.022	−0.143
BH/CPH	0.069	0.776	−0.474	0.000
CPL/CPH	−0.238	0.864	0.085	0.302
D1-2	0.877	−0.382	−0.076	0.116
D1-3	0.935	0.031	0.108	−0.137
D1-13	0.530	−0.474	0.463	−0.281
D2-4	0.876	−0.380	−0.035	0.022
D2-13	0.911	−0.354	0.003	0.073
D3-4	0.934	−0.030	0.135	−0.071
D3-5	0.875	0.205	0.174	−0.071
D3-13	0.961	−0.023	−0.013	−0.164
D4-5	0.860	−0.090	−0.085	−0.088
D4-6	0.899	−0.053	0.117	−0.121
D4-13	0.901	−0.159	−0.162	−0.123
D5-6	0.936	0.052	0.111	−0.119
D5-7	0.895	0.105	0.186	−0.099
D5-8	0.540	0.761	−0.123	0.103
D5-9	0.941	−0.112	−0.091	−0.095
D5-10	0.803	0.527	−0.048	0.060
D6-7	0.950	0.268	0.043	0.028
D6-8	0.924	0.165	−0.117	0.120
D7-9	0.603	−0.373	−0.054	0.183
D7-10	0.903	−0.021	−0.107	−0.070
D7-11	0.846	0.369	0.022	0.168
D7-12	0.849	0.386	0.121	0.137
D8-9	0.607	0.514	0.192	−0.187
D9-10	0.931	0.155	0.109	0.182
D10-11	0.870	0.281	−0.185	0.063
D10-12	0.810	0.289	0.063	0.227
D11-12	0.669	−0.121	0.027	0.092
Contribution rate (%)	56.20	18.68	5.20	2.94

## Data Availability

The data that support the findings of this study are available on request from the corresponding author. The data are not publicly available due to privacy and ethical restrictions.
